# Factor V Leiden G1691A and Prothrombin Gene G20210A Mutations on Pregnancy Outcome

**DOI:** 10.7759/cureus.17185

**Published:** 2021-08-15

**Authors:** Jaskamal Padda, Khizer Khalid, Ayushi Mohan, Sindhu Pokhriyal, Nitya Batra, Gazala Hitawala, Ayden Charlene Cooper, Gutteridge Jean-Charles

**Affiliations:** 1 Internal Medicine, JC Medical Center, Orlando, USA; 2 Internal Medicine, Avalon University School of Medicine, Willemstad, CUW; 3 Internal Medicine, Advent Health & Orlando Health Hospital, Orlando, USA

**Keywords:** prothrombin gene mutation, factor v affecting pregnancy, prothrombin mutation affecting pregnancy, g1691a gene mutation, g20210a gene mutation, factor v leiden mutation

## Abstract

Factor V Leiden (FVL) G1619A mutation and prothrombin gene (PTG) G20210A are the most common inherited thrombophilias. They have been associated with various obstetric complications such as venous thromboembolism, recurrent pregnancy loss, preeclampsia, abruptio placentae, and small for gestational age fetus. The prevalence of these two mutations is 3-15% in Caucasians and is assumed to be far less common in other ethnic populations. However, there have been several controversies regarding advising routine screening of these thrombophilias because of a widely variable strength of association between different ethnic groups, as well as contradictory conclusions by different studies in regards to the association. In this study, the literature was analyzed thoroughly for the effect of FVL G1619A and PTG G20210A mutations on various obstetric outcomes. A review of multiple case-control and prospective studies suggests that despite the availability of robust data on this subject the results remain inconclusive and insubstantial. Further superior quality research, preferably prospective studies, is warranted to conclusively establish this relationship and to enable practitioners to follow a definitive protocol in the screening of various populations for these mutations to achieve an improved pregnancy outcome.

## Introduction and background

Physiological changes during a normal pregnancy result in a hypercoagulable state, which is essential for hemostasis during parturition [1]. The major prothrombotic factors during pregnancy include elevated coagulation factors, decreased natural anticoagulants, and decreased activity of the fibrinolytic pathway [2-4]. The levels of protein C and protein S are reduced with accompanying relative resistance during pregnancy [5-8]. Fibrinolytic activity, which is essential for the lysis of clots and maintaining luminal integrity, is also reduced during pregnancy due to an increase in the levels of the plasminogen activator inhibitor (PAI) [9]. In thrombophilia, this system of physiological changes is disturbed and further affects pregnancy outcomes. Overall, 50% of the patients with thrombosis during pregnancy are found to have an underlying thrombophilia [10].

A missense mutation of factor V Leiden (FVL) and prothrombin gene (PTG) results in a hypercoagulable state [11-16]. The frequency of FVL mutation varies among different racial groups; 6.1% in whites, 0.8% in African Americans, and 1.7% in Hispanic pregnant women in the United States [17]. Its prevalence is 4% in Northern Europe, 7% in Southern Europe, 7.5% in the Middle East, and is extremely rare in Asia, Asia Minor, Africa, and South America (0-0.6%) [18]. The prevalence of the G20210A mutation in the PTG is around 3% in southern Europe, 2% in northern Europe, the Middle East, and the Caucasian population of the United States, whereas it is much rarer (0-0.3%) in Africa, Asia, Asia Minor, South America, and in the non-Caucasian populations of North America [19]. The global prevalence of PTG mutation G20210A is reported from 0% to 15.9% among ethnic groups [20]. Considering that the factor V mutation G1691A and PTG mutation G20210A lead to detrimental pregnancy outcomes, it is important to follow standardized management and screening protocols for these patients.

## Review

Pathophysiology

A normal pregnancy is associated with various changes in the coagulation pathway. These changes include the increase in several clotting factors, decrease in protein S levels, and inhibition of fibrinolysis. There is also a drop in the activity of the activated protein C, which is an important natural anticoagulant. The discussed physiological changes are crucial to minimizing intrapartum blood loss [21].

**Figure 1:**
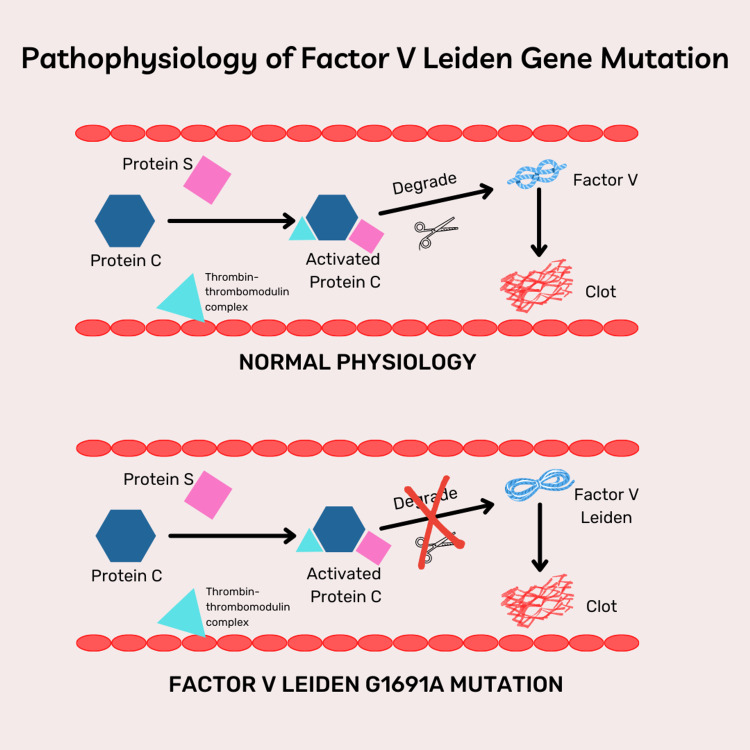
Pathophysiology of factor V Leiden gene mutation. The image was created by one of the authors (Gazala Hitawala, MBBS) [11,12,21,22].

The presence of protein C is important for normal hemostasis. Protein C gets activated after binding to the thrombin-thrombomodulin complex on the endothelial cells with the assistance of the cofactor protein S. Consequently, activated protein C selectively degrades activated factor V (Va) and prevents clot formation [11,21,22]. Factor V G1691A is a mutated protein that is formed via a substitution of guanine for adenine at position 1691, resulting in glutamate being present at amino acid residue 506 (R506Q) instead of arginine [11]. Protein C acts as an anticoagulant that inactivates the factor Va. This disrupts the degradation of factor Va by the activated protein C which results in a hypercoagulable state (Figure 1) [11,12,21,22].

Prothrombin is the precursor of thrombin which is the end product of the coagulation cascade. Thrombin eventually cleaves fibrinogen to fibrin leading to the formation of fibrin clots [23]. Furthermore, thrombin acts on platelets, factor V, factor VIII, factor XIII, and thrombin activatable fibrinolysis inhibitor (TAFI) [24-28]. A single missense mutation on PTG at chromosome 11 leads to the substitution of guanine by adenine at nucleotide position 20210. This mutation is present in the 3’ untranslated region of the PTG and does not affect the structure of the prothrombin molecule [13]. However, this mutation causes increased levels of blood prothrombin, TAFI, along with increased mRNA and protein expression for prothrombin, collectively resulting in a hypercoagulable state [14-16,29]. Although the explicit mechanism through which the PTG G20210A mutation functions is not known, it is reported that TAFI catalyzes the removal of the C-terminal lysine from fibrin. This attenuates the rate of plasmin formation and, subsequently, the rate of fibrinolysis is reduced. Thus, in PTG G20210A mutation, the increased prothrombin results in increased fibrin production while TAFI hinders fibrin degradation, both resulting in an increased risk of thrombosis [30]. The inherited coagulation disorder in factor V G1691A and prothrombin G20210A in pregnancy exacerbates the preexisting hypercoagulable state of pregnancy.

Factor V Leiden and prothrombin mutations and pregnancy loss

Pregnancy loss is one of the principal issues in women’s health. Clinically detected pregnancy loss at any time of gestation occurs in about 15% to 25% of all pregnancies [31]. The definition of recurrent pregnancy loss (RPL) is variable. The commonly used definitions are two or more failed clinical pregnancies as documented by ultrasonography or histopathologic examination and three consecutive pregnancy losses which are not required to be intrauterine [32,33]. It is surmised that less than 5% of women will experience two consecutive pregnancy losses and that only 1% of women will experience three or more [34]. RPL has been associated with many causes such as genetic, anatomical, infectious, endocrine/metabolic, autoimmune, and thrombophilias. Even after exhaustive assessment and evaluation, the definite cause of RPL can be delineated in 50% of patients [35].

Thrombophilia, both acquired and genetic, is one of the established etiologies of pregnancy loss. Two of the most common genetically transmitted mutations include a gain of function mutation in PTG G20210A and FVL mutation G169A which renders it resistant to protein S. These mutations have been remarkably associated with pregnancy loss and other adverse outcomes [36].

A systematic review and meta-analysis involving 89 studies in 2021 showed that women with FVL mutation had higher risks of RPL (odds ratio = 2.44). Similarly, those with PTG mutation were twice as likely to experience RPL (odds ratio = 2.08) [37]. A recent meta-analysis of 19 case-control studies from Middle Eastern countries delineated that the prevalence of FVL mutation was 12.6% in patients with RPL loss compared to 4.9% in controls, with an odds ratio of 2.37 [38]. Yet another review in 2015 involving nine studies showed a statistically significant increased carrier frequency of FVL mutation with an odds ratio of 1.68 in women with recurrent first-trimester fetal losses [39]. In a meta-analysis of 37 case-control studies in 2015, a significant association was found between PTG mutation and RPL (odds ratio = 1.81). This risk was particularly high in Europeans and women older than 29 years [40]. Another meta-analysis of case-control studies in 2011 showed that the odds ratio of RPL in FVL mutation carriers was 2.02. It also suggested that women with G20210A mutations were overall twice as likely to have RPL as those without the mutation (odds ratio = 2.07) [41]. A 2006 meta-analysis performed on 35 studies concluded that FVL mutation was associated with first-trimester pregnancy loss with homozygous and heterozygous odds ratios of 2.71 and 1.68, respectively, and second-trimester loss with an odds ratio of 2.06. In the same study, PTG mutation was also linked to early and late abortion with odds ratios of 2.49 and 2.66, respectively [42]. A study that reviewed five papers in 2002 revealed that women with unexplained stillbirth were six times more likely to be carriers of FVL mutation with an odds ratio of 6.1 [43].

In contrast to the above findings, some studies deduced no relationship between genetic thrombophilia and pregnancy loss. A systematic review of 10 prospective cohort studies from 2010 revealed that factor V mutations were correlated to pregnancy loss (spontaneous miscarriages and stillbirth) with an odds ratio of 1.52, but it failed to show a similar association between prothrombin mutation and pregnancy loss [44]. In 2011, 3.8% out of 4,167 pregnant women tested positive for PTG mutation and deduced no significant relationship between this mutation and pregnancy loss [45]. A multicentric study in 2005 assessing 4,885 gravidas showed no correlation between pregnancy loss and FVL carriage [17]. A study on 1,384 women enrolled in the European Prospective Cohort on Thrombophilia reported that the risk of miscarriages and stillbirths was 35% higher in patients with inherited thrombophilia, including antithrombin, protein C, protein S deficiency, and FVL mutation, but such association was not seen specifically with FVL mutation [46].

Despite having a substantial amount of data on FVL and PTG mutations along with their effects on pregnancy loss, there is still a controversy, and a definite conclusion cannot be made. Various factors such as study designs, research biases, and racial differences have been suggested as the reasons for such mixed and inconclusive findings. Further research is warranted to build a more robust relationship between genetic thrombophilia and pregnancy loss and to establish standardized management and screening guidelines [44].

Factor V Leiden and prothrombin mutations and preeclampsia

Preeclampsia is defined as blood pressure of >/=140/90 mmHg recorded on two occasions four hours apart in a previously normotensive pregnant woman, or blood pressure >/=160/110 mmHg (for instant diagnosis and timely management) [47]. A total of 70 studies done in 30 countries between 1969 to 2019 revealed an overall prevalence rate of preeclampsia to be 6.7% with the highest prevalence found in low- and medium-income regions (11.5%, 95% confidence interval [CI] = 7.8-15.8 and 10.6%, 95% CI = 6.05-16.2, respectively) [48]. The association between FVL and PTG mutations has been extensively studied in various populations. On one hand, studies have shown a strong association between thrombophilia and poor pregnancy outcomes such as preeclampsia [17,42,49-52], but, on the other hand, studies have also failed to depict any relationship between the two [17,53-58].

The prevalence of FVL mutation is more in the European population, especially in the Lebanese [59]. However, a study conducted on Lebanese women between 2003 and 2005 showed that inherited thrombophilia mutations were not significantly associated with the development of severe preeclampsia [59]. Whereas a study conducted on Sudanese women strongly concluded a statistically significant association between the two [60].

Screening for FVL and PTG mutations in women with preeclampsia is also controversial. A case-control study conducted on women with preeclampsia found that more than half had underlying thrombophilia and, therefore, they suggested screening for thrombophilia mutations in pregnant women with preeclampsia [61]. In contrast, another study stated that the heterozygous FVL mutation in mothers is associated with a low risk of venous thromboembolism (VTE), and owing to this finding, they discouraged both universal screening and treatment of low-risk carriers during pregnancy [17].

Whereas various studies support the increased prevalence of FVL and PTG mutation in preeclampsia patients, one study reported that there might possibly be other pathogenic factors playing a role in determining the presentation and complications that these mutations would lead to [62]. More extensive studies are required to determine the role of these other factors to control morbidity and mortality associated with preeclampsia [63].

Factor V Leiden and prothrombin mutations and intrauterine growth restriction

Intrauterine growth restriction (IUGR) is defined as infants whose birth weight is below the 10th percentile of birth weight adjusted for sex and gestational age. IUGR is one of the leading causes of increased antenatal and postnatal morbidity and mortality. It is associated with an increased incidence of stillbirths, neonatal hypoglycemia, hypocalcemia, polycythemia, and respiratory depression [64]. It also leads to adverse long-term sequelae such as developmental delay, learning disability, and chronic diseases such as hypertension, type II diabetes, and peripheral arterial disease [65,66]. The causes of IUGR can be intrinsic to the fetus such as chromosomal and genetic aberrations, anatomical abnormalities, and congenital infections, or it can be extrinsic such as maternal malnutrition, renal disease, preeclampsia, diabetes, thrombophilia, and substance abuse. Unfortunately, its etiology remains clinically elusive in most cases [67].

Thrombophilia predisposes to thrombosis of intervillous spaces which is commonly seen in specimens of placentas derived from women with IUGR newborns [68,69]. Numerous studies have been conducted to analyze the relationship between IUGR and genetic thrombophilia, specifically FVL mutation G1691A and PTG mutation G20210A. However, the results are mixed and inconsistent, with some studies proving significant association between these mutations in mothers and IUGR in infants and others contradicting it [43,44,49,70-77].

A meta-analysis conducted on 32 cohort and case-control studies in 2016 revealed a 40% increase in the risk of IUGR in FVL mutation carriers [70]. A systematic review of 10 studies in 2008 showed a relationship between FVL and IUGR (odds ratio = 2.05) and PTG mutation and IUGR (odds ratio = 2.07) with a particularly strong association in Caucasian women (FVL odds ratio = 4.16, PTG odds ratio = 3.79) [71]. Another meta-analysis in 2004 revealed that FVL mutation is associated with a 4.8-fold increased risk of fetal growth retardation [72]. A systematic review of ten case-control studies from 2004 established a significant association between FVL and IUGR (odds ratio = 2.7) and PTG variant and IUGR (odds ratio = 2.5) [49]. A review of three papers in 2002 concluded that PTG mutation was significantly associated with IUGR (odds ratio = 5.7) [43]. A study in 2002 performed on 65 women whose antepartum course was complicated by mid-trimester IUGR (22-26 weeks) inferred that the prevalence of FVL and PTG mutations was significantly higher among cases compared to controls, that is, 35% versus 3.8% (p < 0.001) for FVL and 15.4 versus 2.0 for PTG (p < 0.05) [73]. Another case-control study in 2001 with 154 participants concluded that FVL and PTG mutations were independently associated with the occurrence of IUGR, with odds ratios of 6.9 and 5.9, respectively [74]. Another meta-analysis of 27 studies in 2009 conferred that the odds ratio (1.23) for the association between FVL and IUGR was significant; however, it was not significant for the PTG mutation [75].

On the contrary, the following studies did not establish a significant association between genetic thrombophilia and IUGR. A systematic review of seven prospective cohort studies from 2010 revealed that the link between maternal FVL and PTG mutations and IUGR was not statistically significant [44]. Another prospective study in 2007, The Glasgow Outcome APC Resistance and Lipid pregnancy study, with a sample size of 4,250 women, did not prove any association between IUGR and FVL mutation [76]. A case-control study in 2002 including 965 women indicated no association between FVL mutation or PTG mutation and occurrence of IUGR in their newborns [77]. Taking everything into account, additional exploration is required to deduce cogent conclusions about IUGR and its association with FVL G1691A and PTG G20210A mutations [49].

Management and screening

FVL G1619A and PTG G20210A mutations have been implicated as the most common inherited thrombophilias which have been associated with various obstetric complications [78]. Although most of these associations remain controversial, VTE, which is a known phenomenon in patients with inherited thrombophilias, has continued to remain one of the leading causes of pulmonary embolism and maternal deaths, especially in developed countries such as the United States [79]. More than 30% of VTE in pregnancy has been accounted for by inherited thrombophilias like FVL G1619A and PTG G20210A mutation. There was an eightfold increase in VTE risk in women with inherited thrombophilias compared to healthy women (hazard ratio = 8.0; 95% CI = 1.2-184) [80,81].

Although this makes universal screening for thrombophilias very tempting, there are several clinical considerations that need to be accounted for. One major setback for widespread screening of these mutations is that even in high-risk ethnic groups, a large percentage of individuals who test positive may be completely healthy with normal pregnancy outcomes. Alfirevic et al., in their study on postnatal screening for thrombophilia in women with severe obstetric complications, found that 39% of women in the control group were completely healthy with normal pregnancy outcomes [82]. Second, the treatment of these mutations encompasses using unfractionated heparin or low-molecular-weight heparin which has many clinical and economic implications. Apart from being expensive and cumbersome, these treatments are also known to be associated with several clinically significant side effects such as bleeding, osteopenia, thrombocytopenia, and allergic reactions. Another important consideration in the obstetric population is the inability to optimally institute neuraxial analgesia in laboring patients on anticoagulants [83].

Finally, the association of FVL G1619A mutation and PTG G20210A mutation with obstetric complications remains a subject of controversy with different studies showing contradictory conclusions. Gris et al. reported improved pregnancy outcomes in women with thrombophilias who had past history of fetal deaths and had been treated with anticoagulants. While there were several questions raised on the design of this study, another nonrandomized cohort study by Warren et al. on the effect of thromboprophylaxis in women with thrombophilias showed no significant improvement in pregnancy outcomes [84,85]. There has been a suggestion that various other confounding factors as well as publication bias may be the reasons why there is an alarming discordance in the results of prospective studies, small case-control studies, and retrospective cohort studies [86,87]. Considering all these studies and review articles, the current stand of various professional bodies may differ slightly but agree on advising against routine screening for inherited thrombophilias in unselected populations. The American College of Obstetricians and Gynecologists supports screening when the results would definitely improve pregnancy outcomes. They advocate screening in women with a personal history of provoked or unprovoked VTE, those with recurrent VTE, and those with a first-degree relative known to have a high-risk thrombophilia. Due to the paucity of data, suggesting a definitive benefit of using anticoagulants to improve pregnancy outcomes in women with RPL, abruptio placenta, preeclampsia, and stillbirths, screening is not advocated in women with these clinical backgrounds. The dosages and length of treatment vary in women depending on the zygosity of the mutations as well as their clinical backgrounds [78,88]. Lastly, there is no universal standardized protocol for diagnosing and managing all women with FVL G1619A and PTG G20210A mutation in pregnancy.

## Conclusions

Pregnancy is a hypercoagulable state. This physiological change is essential for preventing excessive blood loss during childbirth. The inherited mutation of factor V (G1691A) and prothrombin (G20210A) in pregnancy exacerbates the preexisting hypercoagulable state. In this review article, we studied the relationship of FVL and PTG mutation with adverse pregnancy outcomes such as spontaneous abortion, preeclampsia, and IUGR. Thrombophilia, both acquired and genetic, is one of the causes of pregnancy loss. After reviewing a substantial number of studies on FVL and PTG mutations and their effects on pregnancy loss, there is still controversy and definite conclusions regarding miscarriages cannot be made. Similarly, various studies support the increased prevalence of FVL and PTG mutation in preeclampsia patients, yet more extensive studies are required to undoubtedly claim the association and control the morbidity and mortality associated with it. We saw that the chances of adverse events were found to be increased in preeclamptic women with heterozygous FVL mutation compared to preeclamptic women lacking the mutation. In addition, the relationship between the mutations in mothers and IUGR in infants was also found to be mixed and inconsistent, with most studies supporting the association and yet few studies revealed no association between the two. Owing to the present data and taking everything into account, some studies recommend screening for FVL and PTG mutations in women with risk factors, but further research is warranted to build a more robust relationship to establish standardized screening and management protocols.
